# Altered gene expression and ecological divergence in sibling allopolyploids of *Dactylorhiza *(Orchidaceae)

**DOI:** 10.1186/1471-2148-11-113

**Published:** 2011-04-26

**Authors:** Ovidiu Paun, Richard M Bateman, Michael F Fay, Javier A Luna, Justin Moat, Mikael Hedrén, Mark W Chase

**Affiliations:** 1 Department of Systematic and Evolutionary Botany, University of Vienna, Rennweg 14, A-1030 Vienna, Austria; 2 Jodrell Laboratory, Royal Botanic Gardens, Kew, Richmond, Surrey, TW9 3DS, UK; 3 Department of Ecology, Section of Plant Ecology and Systematics, University of Lund, Lund, Sweden

## Abstract

**Background:**

Hybridization and polyploidy are potent forces that have regularly stimulated plant evolution and adaptation. *Dactylorhiza majalis *s.s., *D. traunsteineri *s.l. and *D. ebudensis *are three allopolyploid species of a polyploid complex formed through unidirectional (and, in the first two cases, recurrent) hybridization between the widespread diploids *D. fuchsii *and *D. incarnata*. Differing considerably in geographical extent and ecological tolerance, the three allopolyploids together provide a useful system to explore genomic responses to allopolyploidization and reveal their role in adaptation to contrasting environments.

**Results:**

Analyses of cDNA-AFLPs show a significant increase in the range of gene expression of these allopolyploid lineages, demonstrating higher potential for phenotypic plasticity than is shown by either parent. Moreover, allopolyploid individuals express significantly more gene variants (including novel alleles) than their parents, providing clear evidence of increased biological complexity following allopolyploidization. More genetic mutations seem to have accumulated in the older *D. majalis *compared with the younger *D. traunsteineri *since their respective formation.

**Conclusions:**

Multiple origins of the polyploids contribute to differential patterns of gene expression with a distinct geographic structure. However, several transcripts conserved within each allopolyploid taxon differ between taxa, indicating that habitat preferences shape similar expression patterns in these independently formed tetraploids. Statistical signals separate several transcripts - some of them novel in allopolyploids - that appear correlated with adaptive traits and seem to play a role favouring the persistence of individuals in their native environments. In addition to stabilizing the allopolyploid genome, genetic and epigenetic alterations are key determinants of adaptive success of the new polyploid species after recurrent allopolyploidization events, potentially triggering reproductive isolation between the resulting lineages.

## Background

Recent genomic investigations have uncovered signals of past whole-genome duplications (WGD) across angiosperms, indicating that polyploidy is a common mechanism of genome evolution in flowering plants [1,2]. Over time, polyploids undergo diploidization, eventually behaving like diploids both genetically and cytogenetically, but they retain vestiges of their WGD heritage. The prevalence of WGD across the history of flowering plants suggests that angiosperm evolution proceeds in cycles of genome doubling and subsequent diploidization [2], which have inevitably influenced their evolutionary lineage. WGD may even have been a pivotal evolutionary force during the origin and diversification of angiosperms; by extending functional capacities [3] and creating evolutionary innovation, these WGD events are hypothesized to have been key factors stimulating major phenotypic transitions [4-6]. For example, successive duplications of several key floral organ identity genes from the MADS-box family imply that polyploidy was important for the origin and evolution of the flower, itself a diversity-enhancing feature [7,8]. In addition, a clustering of genome duplications around the Cretaceous-Tertiary (KT) boundary in independent angiosperm lineages indicates that polyploid lineages may be better able to radiate if they are fortunate enough to survive the randomness inherent in mass extinction events [9-11].

Most frequently, WGD arises from meiotic non-reduction followed by the fusion of unreduced gametes, commonly via a 'triploid bridge' [12]. Ramsey & Schemske [12] reported that unreduced gametes are produced at rates on average *ca *50 times higher in hybrids than in non-hybrid lineages. As a result, hybridization and WGD appear to be closely associated in angiosperms, so that evolutionarily successful polyploidization events appear especially prevalent among hybrids [[13], p. 329, [14-16]]. Autopolyploids are nonetheless widespread in nature [17,18], and many remain phenotypically hidden within their diploid parent.

The association of WGD and hybridization may provide several adaptive advantages to an evolutionary lineage. By combining entire parental genomes in the same nucleus, polyploid hybrids potentially benefit at meiotic pairing. They also gain from hybrid vigour and transgressive traits (outside the parental range [19,20]), avoiding problems specific to homoploid hybrids such as segregation and breakdown in F_2 _generations [21]. Additionally, WGD provides allopolyploids with a high degree of post-zygotic reproductive isolation from their diploid relatives [12]. Polyploidy doubles gene number, creating the potential for buffering vital functions (perhaps via homogenization [22]) but also for functional divergence (neofunctionalization), which increases biological complexity [23]. Other proposed advantages of polyploidy relate to relaxation of reproductive system requirements, via loss of self-incompatibility [24] and/or potential for agamospermy [21,25]. By perpetuating the most adaptive hybrid genotypes, allopolyploidy can result in abrupt or even saltational speciation [26]. However, many neopolyploids will still fail to become established because of reproductive failure [27] and/or minority cytotype disadvantage [28]. Stebbins [29] extrapolated from well-studied genera that *ca *30% of all angiosperm species may be functional allopolyploids. In any case, speciation via polyploidy is likely to be a major mode of sympatric speciation in plants [14,30]; recent direct estimates indicate that as many as 15% of angiosperm speciation events are accompanied by WGD [31].

Because of the increased gene and genome dosage, neopolyploids usually suffer from negative effects of expression redundancies, regulatory incompatibilities and meiotic abnormalities [21,32]. Hence, allopolyploidy induces a state of 'genome shock' [33], allowing natural selection to prevent establishment of any maladaptive early-generation polyploid. Exceptions have been also reported: for example wheat synthetic allopolyploids show additivity of expression [34], while in newly synthesized *Gossypium *allopolyploids there is little evidence of genomic shock [35]. However, most of the successful early-generation allopolyploids have to accommodate the two divergent genomes in one nucleus by adjusting organization and function of both genomes through genetic and epigenetic alterations [32,36-38]. Several studies conducted on resynthesized hybrids/allopolyploids (e.g. [39-44]) and wild neopolyploids (e.g. [45-47]) have revealed that hybridization, with or without a shift in ploidy, can quickly result in alterations of gene expression due to gene loss, silencing, subfunctionalization (tissue-specific expression of gene copies) and other non-additive expression patterns. More than one type of alteration has been recorded in most cases of polyploidy that have been studied in sufficient detail. Epigenetic changes, such as gene silencing via DNA methylation change and chromatin remodeling, appear to be a more universal response that, if stably inherited, can rapidly lead to subfunctionalization [36] or to phenotypic differences in recurrent allopolyploids [48].

**Figure 1:**
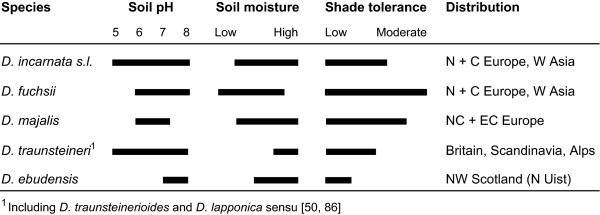
Ecological tolerance and geographical distribution of *Dactylorhiza* allopolyploid species investigated here (based on field observations of RB in Britain, MH in Scandinavia and OP in the Alps and Pyrenees).

Despite our increasing general knowledge of polyploidy and hybridization, information remains limited on the links between genomic responses to allopolyploidization and mechanisms involved in shaping long-term adaptive capacities in natural allopolyploid populations. We lack a detailed understanding of the scope of expression changes and how they function within established natural lineages. For example, we still need to understand the functional correlations between altered gene expression and the development of adaptive phenotypes and to determine their effects on ecological and reproductive isolation and ultimately on evolution of polyploid lineages. We aim here to investigate gene expression alterations in three established, sibling allotetraploid (2*n *= 80) species of the *Dactylorhiza majalis *(Rchb.) P.F.Hunt & Summerh. complex (Orchidaceae: Orchidinae) and to correlate these alterations with their respective ecological preferences (Figure 1).

The widespread allotetraploids *D. majalis *s.s. and *D. traunsteineri *s.l. have each been derived iteratively at different times during the last part of the Quaternary; together with the narrowly endemic *D. ebudensis*, they originated through unidirectional hybridization between the diploids (2*n *= 40) *D. fuchsii *(in all cases the maternal parent) and *D. incarnata *([49] and references therein). Despite their largely shared genetic heritage, the three allotetraploids differ significantly in morphology, ecology and geography (Figure 1). Comparison of the degree of concerted evolution in ITS alleles, the cohesiveness of epigenetic patterns of individuals from different regions [48], and the patterns of morphology and ecological preference together suggest that *D. majalis *s.s. is substantially more derived and genetically homogeneous. It is therefore hypothesized to be the oldest of the three allotetraploids and is presumed to have passed through glacially induced bottlenecks in southern Eurasia [48,49]. It has a comparatively wide ecological tolerance of soil moisture (Figure 1) and presently occurs in damp meadows and fens from western and central Europe to southernmost Scandinavia. In contrast, *D. traunsteineri *s.l. is a more recently evolved set of allotetraploids that is more heterogeneous, both genetically [49-51] and epigenetically [48], and often still maintains both parental ITS alleles. It includes lineages that probably originated post-glacially, and at present shows a more localized and disjunct distribution in northwestern and central Europe. It generally has narrow tolerances of soil moisture, being vulnerable to drought, and grows in fens and marshes. A third allotetraploid, *D. ebudensis*, is a narrow endemic (at present, 99% of known individuals form a single extensive metapopulation in northwestern Scotland); it is considered to be as young as, or younger than, *D. traunsteineri *[48-50]. The coastal dune habitat that confines *D. ebudensis *indicates its relatively narrow substrate tolerances - notably, in the amount of groundwater, its degree of oxygenation and pH (Figure 1). Despite the fact that their distribution ranges partly overlap, the three polyploid taxa have different overall ecological requirements (Figure 1) and are parapatric, rarely co-occurring within the same site.

*Dactylorhiza *offers an excellent model system for studying successful allopolyploidy because: (i) the tetraploid taxa have a recent history of allopolyploidization but have already become well established; (ii) the parental species involved (but not necessarily the exact parental genotypes) remain extant; (iii) *D. majalis *and *D. traunsteneri *originated from the same parental species pair and each has multiple origins, thereby providing natural replicates for study; and (iv) all three taxa differ in habitat preferences, morphology, age and evolutionary history. Overall, this is a suitable study system to uncover mechanisms of (local) adaptation to divergent environments following polyploidy and hybridization, which are assumed to lead to evolutionary diversification and speciation. A recent investigation [48] of these *Dactylorhiza *allopolyploids using MSAP (methylation sensitive amplified polymorphism) demonstrated that divergent selection acts on epigenetic characters and results in differentiation that correlates with adaptation to distinct environments. We show here that similar trends are visible in the expressed patterns of the allopolyploids, and we conclude that ecological divergence between these polyploid lineages resides mainly in quantitative expression differences.

## Methods

### Plant material

Several individuals of each allopolyploid species, together with representatives of the diploid parental species, were sampled from four geographic regions (Table 1): the eastern Alps and Scandinavia, where *D. majalis *and *D. traunsteineri *grow in parapatry; northern Pyrenees, where only *D. majalis *occurs; and Britain, where *D. traunsteineri *s.l. occurs (plus *D. ebudensis *on the remote island of North Uist in northwestern Scotland). Plants were transplanted in the cold glasshouse of the Royal Botanic Gardens, Kew (U.K.), where they were grown in uniform conditions for one year to allow acclimatization prior to leaf sampling. The allopolyploid (but not the diploid) samples included here have been previously analysed epigenetically using the MSAP technique [48]. Before the transcriptomic analyses, each plant was genotyped using nuclear and plastid markers (following [49]) to confirm that it conformed to type (Table 1).

**Table 1:** Details of *Dactylorhiza *samples investigated in the present study.

Ploidy	Species	Latitude/longitude	Collector^1^	ITS alleles (ratio)^2^	Haplo type^2^
**2*x***	***D. fuchsii ***(Druce) Soó	42.829/1.995	C, F, P, L	III (66%): V (33%)	B
		43.212/0.830	C, F, P, L	V (55%): III (45%)	A
		
		46.301/14.435	P	V (60%): III (40%)	A
	
	***D. incarnata ***(L.) Soó	42.829/1.995	C, F, P, L	X (100%)	E
				X (100%)	E

**4*x***	***D. majalis ***(Rchb.) P.F.	42.829/1.995	C, F, P, L	III (66%): V (33%)	B
				III (50%): V (50%)	B
		
	Hunt & Summerh.	47.596/15.294	P	III (50%): V (50%)	C
		47.905/14.166	P	III (50%): V (50%)	A
		
		55.817/12.933	H	III (80%): V (20%)	A
				III (80%): V (20%)	C
	
	***D. traunsteineri ***(Saut. ex Rchb.)	57.417/18.323	H	III (40%): V (30%): X (30%)	C
		
	Verm.	54.265/-0.701	C, F, P	X (50%): V (30%): III (20%)	C
		57.436/-5.801	B	III (90%): X (10%)	C
	
	***D. ebudensis ***(Wief. ex R.M. Bateman & Denholm) P. Delforge	57.663/-7.225	B	V (55%): III (30%): X (15%)	C
				V (55%): III (30%): X (15%)	C

^1^Collectors: B - RM Bateman; C - MW Chase; F - MF Fay; H - M Hedrén; L - L Civeyrel; P - O Paun.

^2^Following [49].

### cDNA-AFLP technique

Being based on mRNA, this method analyzes only the transcribed regions of the genome. However, transcript polymorphism, as identified by cDNA-AFLP, may not necessarily represent expression differences; apart from gene silencing, physical loss and non-synonymous polymorphism (indels, substitutions and rearrangements), synonymous substitutions can also be visible to this method [37,48]. However, due to the relatively recent origin and similar genetic background of the allopolyploids (see above), most of the variation depicted with cDNA-AFLPs is predicted to reflect expression differentiation of parental homeologs rather than polymorphism at the nucleotide level in exons. Gene expression can also be regulated at a later, post-transcriptional stage [52,53]. Parts of this level of regulation could be invisible to the cDNA-AFLP technique. Finally, the cDNA-AFLP fragments will contain, in addition to coding sequence, the untranslated sections (5' and 3' UTRs) that characterize all mRNA strands. Although non-coding, the UTRs are expected to experience selection because they provide signals and binding sites for elements post-transcriptionally affecting mRNA stability or translation [54].

The standard AFLP™ procedure [55] was performed on a pool of cDNAs [37,56] generated from leaves sampled from *Dactylorhiza *plants grown in uniform conditions at RBG Kew as previously reported [48]. The products of 27 primer combinations (of the general type EcoRI AX [+fluorescent dye]-MseI CYZ, where X, Y and Z are different selective nucleotides) were suspended in formamide and run on a capillary sequencer ABI 3100 (Applied Biosystems), together with GeneScan ROX 500 (Applied Biosystems) internal size standard. Blind samples and two replicates (13% of total samples identifying an error rate of 0.8%) were included in all steps to test for contamination and reproducibility [57]. Fragments in the range 50-490 bp were aligned using ABI PRISM GeneScan 2.1 Analysis Software (Applied Biosystems) and visualized, scored and exported as binary presence/absence matrix using Genographer 1.6.

### Data analyses

**Figure 2:**
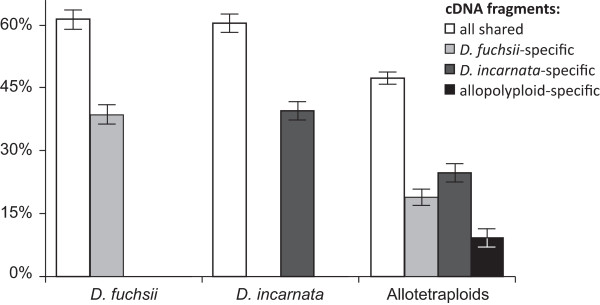
**Average frequencies of cDNA-AFLP fragments in *Dactylorhiza *allotetraploids and their diploid parental species**. White bars indicate shared fragments between the two parentals; light grey bars, markers characteristic of *D. fuchsii*; dark grey, markers characteristic of *D. incarnata*; the black bar shows the unique markers for allopolyploids. The error bars represent standard deviation for mean (see also Table 2).

To investigate the structure of the allopolyploid transcriptome, we first assigned the cDNA-AFLP fragments transcribed from the polyploid genomes according to their status within the diploid individuals. Four categories of fragments have been traced: (i) transcripts characteristic of the maternal species, *D. fuchsii*, (ii) fragments specific for the paternal *D. incarnata*, (iii) fragments shared by the parents, and (iv) markers absent from parental species but present in allopolyploids (i.e. "novel" fragments). The average frequency of the different categories of AFLP fragments present in the allopolyploid genomes is presented as a bar-chart (Figure 2).

In all further analyses, any monomorphic fragments and those present or absent from all but one individual were removed to avoid biased parameter estimates [57]. To visualize the pattern of expression differentiation, we constructed a principal coordinates analysis (PCoA) using the program package NTSYS-pc 2.02h [58]. A matrix of between-individual Dice [59] similarities constructed using the module 'SimQual' was transformed into a scalar form with 'Dcenter', on which we computed the eigenvectors and plotted them using 'Eigen'. The Dice algorithm, which does not treat shared band absence as homologous, was chosen because absence of cDNA-AFLP fragments can result from various causes, possibly involving silencing of a particular homeolog, DNA sequence polymorphism or even developmental differentiation (despite all steps taken to avoid such differentiation). We further estimated the goodness of fit of the PCoA by generating a model distance matrix from the eigenvector matrix (using 'Simint') and comparing it with the original Dice coefficient matrix (with 'Mxcomp' and 1000 permutations). For allopolyploid individuals only, a neighbor-joining (NJ) dendrogram based on the between-individuals genetic distance of Nei & Li [60] was generated and bootstrapped [61] using 1000 replicates with Treecon 1.3b [62].

To identify particular transcripts that are selected within allopolyploid individuals by native environmental conditions, and may therefore play a role favouring their presence in a given landscape, we performed multiple univariate logistic regressions, as implemented in the spatial analysis method (SAM) proposed by Joost et al. [63,64]. This method goes beyond simply identifying genetic loci associated with native ecological conditions, as it also delivers hypotheses regarding the physical factors that could exert a relevant selection pressure in a particular environment. As SAM takes the individual as the reference unit, the analysis functions independently of any notion of population and is largely assumption-free [63]. For this purpose, we have started from a GIS-based ecoclimatic dataset containing 19 bioclimatic parameters from Worldclim (http://www.worldclim.org/bioclim[65]), plus yearly averages for vapour pressure (VapPress, in hPa), percentage cloud cover, number of annual days with ground frost, and maximum sunlight hours (Sunp, in %) for March (III), April (IV), May (V), June (VI) and July (VII), all abstracted from the IWMI Climate and Water Atlas http://waterdata.iwmi.org. We performed an initial check for correlations between the 27 variables with Spearman (two-tailed) bivariate correlations and SPSS 10.0, and the parameters involved in the largest number of correlations were excluded sequentially until no correlation remained. Therefore, for SAM analyses only seven ecoclimatic variables have been retained: BIO1 - annual mean temperature, BIO8 - mean temperature of wettest quarter, BIO13 - precipitation of wettest month, BIO16 - precipitation of wettest quarter, VapPess, and Sunp III (i.e. for March) and SunpVII (i.e. for July). Finally, for the SAM analyses, each particular cDNA-AFLP pattern was retained only once (resulting in a dataset of only 87 cDNA markers), to minimize the number of comparisons performed.

As a measure to control for type 1 errors in multiple comparisons, SAM uses a Bonferroni corrected level of significance [63]. Despite its popularity, this procedure has recently been criticised mainly as being overly stringent [66-68]. Due to the low sample sizes that characterize the expensive cDNA-AFLP method, we expected low statistical power in our analyses. However, as we targeted only coding regions, the chances of identifying signals of selection by chance alone should be much lower than in genomic studies. We chose here not to incorporate Bonferroni corrections but rather to use the alternative false discovery rate procedure (FDR [69]), which controls for the proportion of significant results instead of controlling for single errors. We used the R package QVALUE [70] to adjust *P*-values derived from SAM into corresponding *Q*-values assigning a measure of significance to each of the 609 tests simultaneously performed by SAM. In addition, we used SPSS 10.0 to calculate Spearman's *r*_*s *_for the paired cDNA markers and environmental parameters involved in significant regressions, as a way to report effect size, following the recommendations of [68].

## Results

### Gene expression in diploid and polyploid *Dactylorhiza*

The 27 AFLP primer combinations yielded 305 unambiguous cDNA fragments; of these, 32% were monomorphic among all (diploid and polyploid) individuals analyzed. Within allopolyploids only, 55% of the 291 transcribed fragments were monomorphic. However, all individuals showed distinct transcript profiles. The cDNA-AFLP data matrix and input file for SAM have been lodged in the Dryad Digital Repository at http://dx.doi.org/10.5061/dryad.8795 and it is also available as Additional file 1.

Across the cDNA-AFLP dataset there was a remarkably similar number of fragments characteristic of either parent: 74 markers were specific for *D. fuchsii *and 75 for *D. incarnata*, which contributed to a total transcript differentiation between the diploid parental species of 58% (Table 2, Figure 2).

**Table 2:** Transcriptomic patterns in *Dactylorhiza *samples investigated in the present study.

			cDNA-AFLP				
			
Ploidy	Species	Region	N_ind_	N_frag_	F%	I%	A%
**2*x***	***D. fuchsii***	Pyrenees	1	168	40%	-	-
			1	162	36%	-	-
		
		Alps	1	177	40%	-	-
	
	***D. incarnata***	Pyrenees	2	181	-	41%	-
				177	-	38%	-

**4*x***	***D. majalis***	Pyrenees	2	234	24%	22%	9%
				218	18%	22%	10%
		
		Alps	1	239	19%	26%	10%
			1	231	19%	26%	7%
		
		Scandinavia	2	231	18%	27%	8%
				227	19%	27%	7%
	
	***D. traunsteineri***	Scandinavia	1	226	19%	27%	7%
		
		Britain	1	221	19%	24%	9%
			1	211	17%	26%	8%
	
	***D. ebudensis***	Britain	2	223	17%	23%	13%
				223	18%	22%	13%

N_ind _- number of individuals analyzed. N_frag _- number of fragments per profile; F%, I%, A% - percentage of diagnostic transcripts of *D. fuchsii*, *D. incarnata *and allopolyploids.

The allopolyploid transcriptome (Table 2, Figure 2) consists of 17-24% maternal (i.e. of *D. fuchsii *origin), 22-27% paternal (i.e. inherited from *D. incarnata*) and 45-50% non-specific transcripts (i.e. shared by the two parents). Many of the inherited patterns are represented by fixed transcripts within the allopolyploids. Approximately 9% of the cDNA markers in allopolyploid taxa have been inherited from the maternal progenitor and are expressed by each allotetraploid individual, and 16% are fixed within the allopolyploids and have been inherited from the paternal parent.

As much as 7-13% of allopolyploid transcripts appear to be novel (Table 2, Figure 2), but none represents a repeatable event across all of the inferred independent allopolyploidization events. Finally, relatively few fragments (5.3% from *D. fuchsii *and 2.3% from *D. incarnata*) occur in parental species but are missing from all allopolyploids; these absences may represent repeatable events of gene loss and silencing (although they may also include synonymous restriction site changes). A much larger fraction of the parental transcriptome has been silenced/lost in at least one polyploid individual (32% from the *D. fuchsii *transcript pool and 22% from *D. incarnata*).

### Taxonomic and geographic transcriptomic differentiation

**Figure 3:**
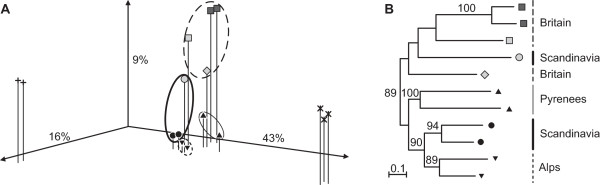
**Expression patterns in *Dactylorhiza *allotetraploids and their diploid parental species**. **A**. Principal coordinates analysis (PCoA; goodness of fit 0.95 at *P *= 0.001) of the Dice similarity matrix [54] between allotetraploid *Dactylorhiza *individuals (filled symbols) and representatives of their diploid parents (unfilled symbols), based on cDNA-AFLP phenotypes. Black-filled symbols, *D. majalis *s.s.; light grey, *D. traunsteineri *s.l.; dark grey, *D. ebudensis*; crosses, *D. incarnata*; stars, *D. fuchsii*. For allopolyploids, shapes of symbols and lines denote geographic provenance of samples: dotted line and inverted triangles, Alps; thick line and circles, Scandinavia; thin line and triangles, Pyrenees; dashed line, Britain with rhombus and squares indicating samples from Yorkshire and Scotland, respectively. The three ordination factors together explain 68% of the total variation present in the cDNA-AFLP data matrix. **B**. Neighbor-joining (NJ) dendrogram based on Nei & Li distances [55] among cDNA-AFLP phenotypes of allopolyploid *Dactylorhiza *individuals. Numbers above branches are NJ bootstrap percentages (1000 replicates) higher than 70. Symbols follow Figure 3A.

The three-dimensional PCoA (Figure 3A) of cDNA-AFLP phenotypes shows clear separation of the two diploid species and of the allopolyploids, which occupy an intermediate position between the parental species along the first axis; this describes a substantial 43% of the variation present in the data matrix. The second axis of variation (16% of the signal) separates the representatives of diploid parental species and the allopolyploid group, but places the latter outside the parental range. The three allopolyploid species are clearly separated from each other by the third axis (containing 9% of the signal), whereas the combined information of the three factors separates the four corresponding geographical provenances for the allopolyploids. The goodness-of-fit analysis of the scatter-plot indicated a matrix correlation value of r = 0.95 at *P *= 0.001 (one-tailed probability).

In the NJ analysis of allopolyploid individuals (Figure 3B), two major phylogroups are separated with 89% bootstrap support (BS): one formed by accessions of *D. majalis *and the other formed by *D. traunsteineri *plus the phenotypically similar *D. ebudensis*. Therefore, *D. traunsteineri *appears as a paraphyletic group, although this relationship receives low bootstrap support (BS < 70%). Within *D. majalis*, the Scandinavian and Alpine accessions studied here form a well-supported subgroup (BS 90%).

### Polyploid adaptive segregation

The univariate logistic regressions corresponding to the Wald test (as implemented in SAM) reached the maximum number of iterations before the maximum likelihood equation had been solved; they were therefore discarded, following the recommendations of Joost *et al. *[63,64]. The alternative test implemented in SAM, the likelihood ratio or G statistical test, identified 115 (19% of the total) regressions with *P*-values lower than 0.05. However, after adjusting the level of significance of multiple tests with the FDR procedure, only 39 (6% of the total) regressions remained significant with *Q*-values of significance lower than 0.05 (Table 3). Due to partial correlation between the environmental parameters, the 39 significant correlations involve 27 adaptive markers. Notably, several of these transcripts represent novel fragments in allopolyploids (Table 3).

**Table 3:** Transcriptomic patterns under environmental selection.

cDNA fragment^1^	Fragment present always and only in^2,3^	Environmental factor^4^	G test		Spearman test	
			
			*P*-value	*Q*-value	*r*_s_	*P*-value
M41	*Dm*	BIO8	0.000099	0.0103	0.87	0.0005
		SunpVII	0.0022	0.027	0.64	0.03

M16	*Dm *from Alps and Scandinavia	BIO8	0.00015	0.0103	0.84	0.001

M59	Britain (*Dt *and *De*)	BIO8	0.00015	0.0103	-0.84	0.001
		SunpVII	0.00015	0.0103	-0.85	0.001

M38	*Dm *from Alps and	VapPres	0.00015	0.0103	-0.84	0.001
	Scandinavia	SunpIII	0.00015	0.0103	0.85	0.001

M12	-	BIO1	0.00033	0.0108	-0.78	0.005

M82	-	BIO8	0.00033	0.0108	-0.78	0.005

M4	Scandinavia (*Dm *and *Dt*)	BIO13, BIO16	0.00033	0.0108	-0.78	0.005

M37	-	SunpIII	0.00033	0.0108	0.79	0.004

M43	-	SunpVII	0.00033	0.0108	0.79	0.004

M58	Scotland (*Dt *and *De*)	SunpVII	0.00033	0.0108	-0.79	0.004

M18	-	BIO8	0.00043	0.013	0.84	0.001

M62	-	SunpVII	0.0011	0.017	-0.79	0.004

M1	*Dt *from Britain	SunpIII	0.0012	0.017	-0.68	0.02

M33	Pyrenees (*Dm*)	SunpIII, SunpVII, BIO1	0.0012	0.017	0.68	0.02

M65	All except Pyrenees	SunpIII, SunpVII, BIO1	0.0012	0.017	-0.68	0.02

M10	Alps (*Dm*)	BIO1, VapPres	0.0012	0.017	-0.68	0.02

M80	All except Alps	BIO1, VapPres	0.0012	0.017	0.68	0.02

M3	*Dm *from Scandinavia	BIO8	0.0012	0.017	0.68	0.02

M84	-	BIO13, BIO16	0.0012	0.017	-0.68	0.02

M44	All except *De*	VapPres	0.0012	0.017	-0.68	0.02

M57	*De*	VapPres	0.0012	0.017	0.68	0.02

M29	-	VapPres	0.0015	0.019	-0.75	0.008

M39	-	SunpVII	0.0015	0.019	0.76	0.007
		VapPres	0.0044	0.048	-0.79	0.004

M61	-	SunpVII	0.0015	0.019	-0.76	0.007

M36	-	SunpIII	0.0022	0.027	0.72	0.01

M20	-	BIO8	0.0041	0.045	0.79	0.004

M32	-	BIO8	0.0041	0.045	0.79	0.004

Details of the 27 cDNA-AFLP patterns most likely to drive adaptation to divergent environments, together with the corresponding environmental variables involved, as indicated by the likelihood ratio (G) test implemented in SAM [58]. Significance measurements for the regressions have been adjusted for multiple testing into *Q*-values according to Storey [70]. We report here only relationships with *Q *values < 0.05. As a way to report effect size for each significant regression we also report Spearman's *r*_s_, following recommendations of Nakagawa [68]. Several environmental parameters show a degree of correlation, complicating attempts to identify the variable that may exert greatest selective pressure. The underlined markers are novel in allopolyploids (i.e. absent from the parents).

^1^For details on cDNA-AFLP markers see http://dx.doi.org/10.5061/dryad.8795 or additional file 1.

^2^The patterns reported in this column refer only to polyploidy individuals.

^3^*Dm *- *D. majalis*; *De *- *D. ebudensis*; *Dt *- *D. traunsteineri*.

^4^BIO1 - annual mean temperature; BIO8 - mean temperature of wettest quarter; BIO13 - precipitation of wettest month; BIO16 - precipitation of wettest quarter; VapPres - yearly averages for vapour pressure (in hPa); SunpIII - % of maximum sunlight hours in March; SunpVII - % of maximum sunlight hours in July.

## Discussion

The cDNA-AFLP technique provides a useful tool for investigating gene expression alterations following genome doubling and/or hybridization [45], especially in non-model systems that lack well-developed genomic resources. However, its expense and the type of data produced (i.e. anonymous and not quantitative) render the approach less desirable when it is compared with more recently developed technologies such as digital transcriptomics (mRNA-seq [71]).

In the present study, we detected a high level of transcript differentiation (Table 2, Figure 2) between the two diploid progenitor species, which proved instrumental for further analyzing the transcriptome of allopolyploids. The high differentiation between the parental transcriptomes is consistent with several studies based on other molecular markers, which have recorded wide genetic divergence between *D. fuchsii *and *D. incarnata *[49,72]. Phylogenetic analyses of *Dactylorhiza *using nrDNA sequences of both internal and external transcribed spacers and the intron of the plastid gene *rpl16 *also indicated that *D. fuchsii *and *D. incarnata *are well differentiated and placed in separate, distinct clades [49,73-75].

### Allopolyploid transcriptomic content

Our investigation of the transcriptomic composition of sibling allopolyploid *Dactylorhiza *species (Table 2, Figure 2) indicated that, although allopolyploid transcriptomes are largely expressing parental genes, a significant degree of novelty also exists. Given the age of the polyploidization events (most probably few to several thousand years), the presence of transcripts specific for allotetraploids and the absence of some markers specific for either parent may reflect post-allopolyploidization evolution of the parental species or incomplete sampling within parental groups. However, at least some of the novel patterns may have resulted from recombination between parental homeologs or from accumulation of mutations in sequences of polyploids [38,76]. In the (most probably) hundreds of generations since the allopolyploid formation, such novel transcripts must have already proved adaptive in their genomic (i.e. internal) and environmental (i.e. external) context and have been retained by natural selection (see also below). The novel fragments are much more common in these established allotetraploid lineages of *Dactylorhiza*, compared with the rather limited proportion (*ca *1%) of non-additive transcripts identified using the same method in the *Tragopogon miscellus *neopolyploids (less than 80 years old; [45]). In contrast, the percentage of parental fragments that are missing in the allopolyploids resembles figures reported in other systems; for example, the transcriptome of allotetraploid cotton lacks *ca *25% of parental alleles [77]. Importantly, our data clearly indicate the stochastic nature of all these genomic alterations, which is particularly visible among the independently formed allopolyploids.

We further observe an obvious tendency for allopolyploids to transcribe more fragments characteristic of the paternal *D. incarnata *than of the maternal *D. fuchsii *genome (significant at *P *< 0.001, paired *t*-test), despite the similar number of diagnostic transcripts exhibited by the diploid species (the difference in the number of specific transcripts in progenitor species was rejected at *P *= 0.184, independent samples *t*-test). This pattern contradicts the strong maternal bias reliably evident in most phenotypes of terrestrial orchids [78]. It may indicate that *D. fuchsii *genes are more often silenced/lost or that the rate of evolution of the maternal genome of allopolyploids is higher and more often results in novel cDNA fragments. This pattern contrasts with results in other systems - for example, in reciprocal hybrids of *Oryza *[43] - where allelic bias of gene expression in hybrids has been found to simply correlate with parental differences. On the other hand, a weak maternal expression dominance has been observed in the neopolyploid *Spartina anglica *[47].

Considering the fact that *D. incarnata *is a far more genetically homogeneous species than *D. fuchsii *[49,50,72,79], an alternative explanation is that analyzed *D. incarnata *individuals are genetically more similar to the actual paternal progenitors of the allopolyploids than those on the maternal side. The difference between proportions of parent-specific fragments inherited in *Dactylorhiza *allopolyploids is generally consistent with genomic data [72]. However, at some loci, the converse trend is evident; ITS nuclear rDNA copies are generally converted towards the maternal parent in *Dactylorhiza *allopolyploids (with few exceptions, notably *D. sphagnicola*; [49]). Studies in other systems have shown that the relationship between gene copy number and expression is not always positive; for example, in *Tragopogon *neoploids, rDNA repeats of *T. dubius *origin are highly expressed and dominate rDNA transcription, even though homogenization has substantially reduced their copy number [80].

Another interesting aspect of the data obtained is that the number of cDNA-AFLP fragments transcribed in allopolyploid individuals (Table 2) was significantly higher compared with either progenitor lineage (*P *< 0.001, independent samples *t*-test). *Dactylorhiza majalis *had on average 33% more fragments than either diploid parental species, *D. traunsteineri *27% more, and *D. ebudensis *29% more; these figures may indicate an increase in the number of gene variants expressed in the allopolyploids. The difference observed is very similar with the pattern obtained comparing with RNA-seq the natural allopolyploid *Glycine dolichocarpa *and its diploid progenitors [81]. However, this pattern contradicts the widely held expectation that closely related organisms, independent of their ploidy, will express a similar number of genes in a given tissue in a shared environment at a particular moment in time - the underlying logic being that they are required to fulfil a similar number of functions. Hence, an increase in the number of fragments transcribed in allopolyploids provides evidence of an increase in complexity involving more extensive regulatory networks, subfunctionalization of expression between different leaf tissues, and/or neofunctionalization [23,82]. Alternatively, both copies of duplicated genes may remain active and retain their original function over a long evolutionary time in polyploids if the relative gene-product stoichiometry is essential for appropriate cellular function or if the genes involved experience allele-dosage effects [83] that provide selective advantages. Many genes present in the genomes of extant angiosperms appear to have originated as a result of ancient polyploidization, especially many of those involved in development, transcriptional regulation and signaling [6,23,84]. The increase in number of such key genes is thought to have been of major importance for the evolution of biological complexity and the introduction of new phenotypic architecture in evolution [85].

Further, there was a marginally significant difference (*P *= 0.045, independent samples *t*-test) in the number of different transcripts amplified in individuals of the older allopolyploid *D. majalis *(average 230) and the cDNA markers in the younger *D. traunsteineri *and *D. ebudensis *(average 221). This trend may indicate that, with time, homeologs are re-activated and/or undergo more divergence via subfunctionalization or neofunctionalization. Such a pattern corroborates results of epigenetic investigations [86], revealing more methylation novelties in the *D. traunsteineri *genome than in *D. majalis*, which may be gradually reverting towards an epigenetic state closer to those of the parents.

### Increased transcriptomic variation within allopolyploids

In addition to significantly more transcripts per profile, allopolyploid lineages show an extended range of transcript variability (illustrated by the increased space occupied by the allopolyploids in the transcriptomic landscape; Figure 3A). For example, diploid *D. fuchsii *samples from the eastern Alps and northern Pyrenees differ at only 12% of the expressed loci, but samples of *D. majalis *from the same regions differ at 29% of their (more numerous) loci. Products of clearly different polyploidization events can be even more divergent; there is up to 40% transcript difference between individuals of *D. majalis *and *D. ebudensis*. This may indicate a greater potential for expression plasticity of the allopolyploids resulting in phenotypic variability exceeding that found in either parent, thus indicating an adaptive advantage. However, this pattern is not mirrored in genomic AFLP data [72], where the differences between samples of parental species, and between samples from different allotetraploids, occupy a narrower interval (e.g. 43% differentiation within *D. fuchsii *and 49% between *D. traunsteineri *and *D. majalis*; see table 1 in [72]).

### Taxonomic and geographic differentiation of the allopolyploid transcriptomes

The multiple independent origins of the tetraploids *D. majalis *and *D. traunsteineri *contribute to differential patterns of gene expression that show some geographic structure (Figure 3A). Several of the investigated transcripts are conserved within each allopolyploid taxon but are variable between taxa. The narrow-endemic *Dactylorhiza ebudensis *seems to integrate within the gene pool of *D. traunsteineri *(Figure 3B), but more studies are necessary for any taxonomic conclusions. At least 9% of the transcript data (the third vector obtained from the PCoA; Figure 3A) and the patterns within NJ (Figure 3B) clearly discriminate between the allotetraploid taxa. In contrast, the results of genomic AFLPs (mostly non-coding) do not provide clear clustering of allopolyploids relative to either geography or taxonomy [72]. This may indicate that habitat shapes similar expression patterns in some (but not all) of the independent allopolyploidization events, perhaps operating via epigenetic alterations under the effect of specific environmental pressures [48]. Given the present data, we cannot exclude the alternative hypothesis that the three allotetraploids may have been formed *in situ *by different parental types from within the diploid species, and thereby inherited distinct gene complexes already well-attuned to the appropriate habitats [87]. However, epigenetic investigations contradict the latter hypothesis [86].

### Polyploid adaptive segregation

Allopolyploidy can have broad-scale effects on gene regulation and developmental processes; it is a source of novel phenotypes capable of prompting ecological diversification and new niche invasion [30]. In contrast with most other polyploid complexes, allotetraploid *Dactylorhiza *species do not exhibit broader geographical distribution ranges than their parents. The diploid species have extensive overall distributions, so that natural barriers (e.g. the Mediterranean and Nordic Seas) are likely to act as equally effective physical constraints on both diploids and polyploids, most probably through limiting distribution of appropriate mycorrhizae (e.g. [88]). However, allopolyploid lineages may have broader amplitudes in at least some ecological parameters than their individual diploid progenitors; for example, they apparently tolerate greater fluctuations in soil moisture than does *D. incarnata*. Their increased overall gene expression diversity and more numerous transcript variants within individuals are likely to contribute to more robust/complex regulatory networks and to heterosis, which may facilitate adaptation to novel conditions. Expression of duplicate genes diverges rapidly in response to changes in environmental (both abiotic and biotic) stresses, but relatively slowly in response to developmental changes that are associated with complex biological networks [89].

Polyploidy provides a vast reservoir of new alleles for mutation and selection, and hence is a prominent mechanism of speciation [14,30]. In general, WGD will immediately provide an allopolyploid with a high degree of postzygotic reproductive isolation from its diploid progenitors [12]. However, the products of recurrent allopolyploidization can suffer from a lack of isolation from each other. Given that they have the same ploidy and similar genomic heritage, maintenance of distinctiveness between independently formed sibling *Dactylorhiza *allopolyploids is likely to prove difficult in the face of substantial gene flow, though it is obviously possible. The most likely explanation is that, in *Dactylorhiza*, apparently weak reproductive isolation between allopolyploids is reinforced by considerable ecological divergence, perhaps inherited partly from distinct parental lines and partly via gene-expression differentiation. However, the balance between these two contrasting factors remains contentious, even among the present authors.

**Figure 4:**
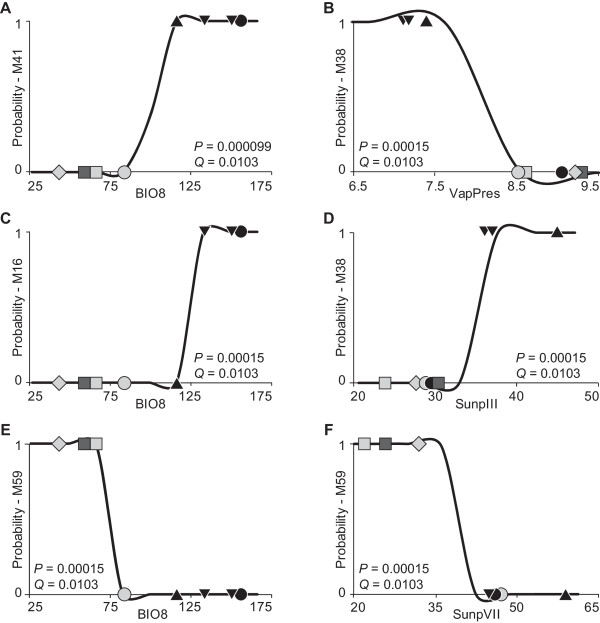
**Expressed patterns under environmental divergent selection**. Most relevant loci under environmental selection as indicated by the likelihood ratio (G) test implemented in SAM [58]. The X-axes contains information from ecoclimatic variables, the Y-axes gives information from the molecular data. Lines indicate the predicted graphs of the logistic sigmoid functions corresponding to relevant pairs of transcriptomic markers and their associated environmental variable (Table 3). The level of significance of the obtained regression is given for each example as both *P*-value (uncorrected significance) and *Q*-value (corrected significance with the FDR method in the context of multiple testing [69]). Symbols indicate the observed transcriptomic data of individuals for the corresponding value of the investigated ecoclimatic parameter. The shape and infill colour of the symbols follow Figure 3A. Some loci correlated with more than one ecoclimatic variable (e.g., loci M38 and M59, see also Table 3). BIO8 - mean temperature of the wettest quarter (measured in °C *10); VapPres - vapour pressure (in hPa); SunpIII and SunpVII - % of sunlight hours in March and, respective, July.

**Figure 5:**
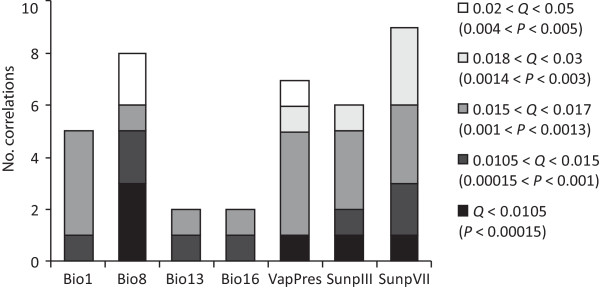
**Environmental variables that exert a relevant selective pressure**. Bar chart summarising the significant regressions obtained with SAM [58] (see also Table 3). The level of significance (*Q*) is adjusted from *P*-values to control for type 1 errors with the FDR method [69]; only relationships with *Q *< 0.05 are retained. Some markers are associated with several ecological variables (Table 3).

In any case, our analyses identify several of the transcriptomic patterns as being significantly correlated with native environmental conditions (Table 3, Figure 4). These are soon rendered adaptive, triggering and then maintaining ecological segregation between the allopolyploids. In this respect, BIO8 (mean temperature of the wettest quarter) appears to be one of the most relevant environmental factors exerting selective pressures among *Dactylorhiza *allopolyploids - it is involved in numerous significant regressions (Figure 5), including the most significant (Table 3). This conclusion receives further support from previous results of epigenetic investigations in this system [48]. BIO8 separates *D. majalis *from *D. traunsteineri *and *D. ebudensis *(Figure 4) and appears to be the primary epigenetic tool of divergent selection increasing their differentiation. Given the marsh-like habitats occupied by *Dactylorhiza *allopolyploids in general, we can easily find the biological interpretation of the adaptive function of a water- and temperature-related climatic parameter. Other important selective pressures seem to be exerted by VapPres (vapour pressure) and SunpVII (% of sunlight hours in July) (Table 3, Figures 4 and 5).

In general, there has been insufficient exploration of levels of gene-flow among co-occurring *Dactylorhiza *allotetraploids. In England and Wales, there is ostensibly a surprisingly narrow 'hybrid zone' occupied by the northern tetraploids *D. purpurella *and *D. traunsteineri *(as narrowly circumscribed in the British Isles by [50]) and the southern tetraploid *D. praetermissa*, which more closely resembles *D. majalis *genetically (e.g. [49]). The hybrid zone coincides with the Weichselian glacial maximum [50,90], suggesting an edaphic influence on their distributions in addition to latitude-related climatic clines. There is growing evidence of gradual southward expansion of *D. purpurella *and northward expansion of *D. praetermissa *into each others territory, with concomitant introgression [50], particularly in dune slacks and quarries [90]. Such populations merit detailed (epi)genetic and autecological investigation to determine the extent of gene flow and identify which factors have precluded more rapid migration.

## Conclusions

Neutral genomic differentiation between *Dactylorhiza *allopolyploids is rarely clearly diagnosable [48-50,72]. This ambiguity contrasts with the surprisingly distinct expression patterns observed (Figure 3), although coding regions are generally expected to evolve much more slowly, and their epigenetic divergence is clear [48]. In the light of present and previous results [48], physical (genetic) diversification *per se *may be less relevant for allopolyploids; divergence between them may instead reside in quantitative partitioning of expression patterns, mainly via epigenetic changes that affect the level of expression of individual genes. Indeed, expression levels of a gene alone can determine phenotypes that contribute to the natural variation on which selection operates [91]. The key extrinsic factor responsible for the environmental selective pressures that are shaping adaptive expression patterns in *Dactylorhiza *allopolyploids seems to be a combination of water availability and temperature, perhaps in addition to pH and associated soil conditions. This conclusion is not surprising, given the moist equable habitats that these lineages usually occupy. Further studies of gene expression, combined with detailed exploration of the *in situ *ecological tolerances of diploid and allotetraploid taxa, should help to better understand the significance of iterative polyploid evolution and to identify the exact functions that are differently regulated in sibling lineages following recurrent hybridization and WGD.

## Authors' contributions

OP, RB, MF, MH and MC designed the study and participated in collecting the samples. JL genotyped samples at various loci prior to the cDNA analyses. OP performed the cDNA studies and most of the analyses. JM extracted the environmental data-points for the sampling localities from WorldClim. OP drafted the paper while RB provided the ecological context. OP and RB revised the manuscript. All authors read and approved the final manuscript.

## Supplementary Material

Additional file 1 **The cDNA-AFLP data matrix and input file for SAM **[58]. Excel file with one worksheet comprising the cDNA-AFLP binary data for diploid and polyploid individuals analysed here. A second worksheet includes the input for SAM [58]. Click here for file
